# Primary care coverage and individual health: evidence from a likelihood model using biomarkers in Brazil

**DOI:** 10.1186/s12913-021-07329-9

**Published:** 2021-12-04

**Authors:** Fernando Antonio Slaibe Postali, Maria Dolores Montoya Diaz, Natalia Nunes Ferreira-Batista, Adriano Dutra Teixeira, Rodrigo Moreno-Serra

**Affiliations:** 1 Department of Economics, University of Sao Paulo, Avenida Prof. Luciano Gualberto, 908, São Paulo, SP 5508-010 Brazil; 2 Fipe-The Institute of Economic Research Foundation, São Paulo, Brazil; 3 Department of Economics, Insper-Institute of Education and Research, São Paulo, Brazil; 4 Centre for Health Economics, University of York, York, UK

**Keywords:** Primary care, Biomarkers, Probit, Impact evaluation, Universal health care, Low- to middle-income countries

## Abstract

**Background:**

Although the use of biomarkers to assess health outcomes has recently gained momentum, literature is still scarce for low- to middle-income countries. This paper explores the relationship between primary care coverage and individual health in Brazil using a dataset of blood-based biomarkers collected by the Brazilian National Health Survey. Both survey data and laboratory results were crossed with coverage data from the Family Health Strategy (ESF) program, the most important primary care program in Brazil; the coverage measures aim to capture both direct (household) and indirect (spill-over) effects.

**Methods:**

The empirical strategy used a probit model to estimate the relationship between ESF program coverage and the likelihood of abnormal biomarker levels while controlling for a rich set of individual and household characteristics based on data from the national survey.

**Results:**

Household ESF coverage was associated with a lower likelihood of abnormal results for biomarkers related to anemia (marginal effect between − 2.16 and − 2.18 percentage points), kidney failure (between − 1.01 and − 1.19 p.p.), and arterial hypertension (between − 1.48 and − 1.64 p.p). The likelihood of abnormal levels of white blood cells and thrombocytes was negatively related to primary care coverage (marginal effect between − 1.8 and − 2 p.p.). The spillover effects were relevant for kidney failure and arterial hypertension, depending on the regional level. Although not sensitive to household coverage, diabetes mellitus was negatively associated with the state supply of primary care, and abnormal cholesterol levels did not present any relationship with ESF program coverage.

**Conclusions:**

The presence of spillover effects of ESF program coverage regarding these conditions reveals that the strengthening of primary care by increasing the household registration and the regional density of ESF teams is an efficient strategy to address important comorbidities.

**Supplementary Information:**

The online version contains supplementary material available at 10.1186/s12913-021-07329-9.

## Background

The Family Health Strategy (ESF) program is the largest primary health care program in Brazil. Launched in 1993, the ESF program has, over the past two decades, spread gradually across Brazilian municipalities as part of a strategy to strengthen basic health care attention. The program is structured by health teams comprising one physician, one nurse, one auxiliary nurse, and four to six community health agents. Community health workers are responsible for periodic home visits to local residents to assess their general health conditions and, if necessary, to give them referrals to outpatient clinics, where the rest of the team members are based.

Most of the existing literature on the topic assesses the impact of the ESF program [1], finding evidence that it has succeeded in reducing infant and/or maternal mortality [2–7], the mortality rates of some diseases and comorbidities [8–11], and admission to outpatient clinics and hospitals due to primary care-sensitive conditions [12–15]. However, a gap has been found in this literature, as the channels whereby the ESF program’s actions affect individual health have not received deserved attention.

Up to 2008, every 5 years, the Brazilian Institute for Geography and Statistics (IBGE) included a special supplement on health in its Annual National Household Sample Survey (PNAD) with specific questions regarding the health of the population. From 2013 onward, this special supplement on health was converted into a broader separate survey called the National Health Survey (PNS). It contained more than 700 questions grouped into 21 themes, including children’s health, women’s and elderly people’s health, chronic diseases, oral health, eating habits, smoking habits, physical activity, health insurance coverage, search for medical services, hospital or outpatient clinics, household income, visits by community agents (including ESF program), and self-assessment of health, among other themes [16].^1^

The PNS 2013 collected anthropometric measures (weight, height, and waist circumference) from household representatives and measured their blood pressure. A subsample of surveyed household representatives was assigned to attend a laboratory clinic to collect biological material (blood and urine) to be tested for some biomarkers. In total, 8952 adult individuals over the age of 18 years had their biological material collected. The following laboratory tests were performed [18]: a hemogram and its components (red blood cell, white blood cell and platelet counts); glycated hemoglobin and estimated average blood glucose levels; total cholesterol levels and fractions (HDL and LDL); serial creatinine levels (from which the glomerular filtration rate was calculated [19]); and a serology test for dengue (IgG). The excretion of creatinine, potassium, salt, and sodium was estimated in the urine samples [18]. The data of individual tests were made publicly available on the Oswaldo Cruz Foundation’s PNS website [20].^2^ The information on biomarkers in this dataset^3^ represents a unique opportunity to contribute to the understanding of the individual channels of the ESF program’s impact on the prevalence of some diseases.

Most of the studies with biomarkers and health interventions focus on high-income countries and cover a wide range of purposes and methods [23–32]. This study aimed to contribute to a better understanding of the channels by which the ESF program affects individual health in Brazil using the results of the laboratory tests described above. The main goal was to investigate whether there is any relationship between primary health care and the biomarkers of the examined individuals. To the best of our knowledge, this is the first study that adopts this strategy to try to unveil the mechanisms through which primary care coverage may impact health in Brazil.

## Methods

The set of diseases and comorbidities assessed in this study was limited by the blood-based biomarkers and the exams contained in the laboratory tests, focusing on conditions that are sensitive to primary care [33]. The following health problems were investigated: leukopenia, leukocytosis, diabetes mellitus, hypercholesterolemia, kidney failure*,* anemia, thrombocytopenia, and arterial hypertension. Charts S1 and S2 in the Additional File 1 list the corresponding biomarkers, their ranges for abnormal results and the proportion of tests according to the defined ranges.

As the laboratory results were made available separately from the PNS 2013 survey, it was necessary to adopt a procedure for merging both datasets to associate each biomarker level with the corresponding interviewed individual. To this end, an identification key was built based on the following information presented in both datasets: weight, height, sex, age, ethnicity, health self-evaluation, and level of education. The identification keys were complemented with specific questions (doctor appointments, nourishment, and health insurance) to maximize the key variability and the percentage of matches. This key proved to have a success rate of 99.8%: from the 8952 individuals tested, only 17 did not match.

As the PNS allowed for the identification of the state and, if applicable, the metropolitan area that each surveyed household was in, it was possible to associate each laboratory result to the regional supply of the ESF teams.^4^ This represented an opportunity to assess potential positive externalities of the program. The hypothesis to be tested was whether a higher supply of health primary care services, measured by the number of the ESF teams by 10,000 inhabitants near the household, implied a more effective result because nonregistered individuals could benefit indirectly from spillover effects produced by health interventions [34, 35]. Therefore, in addition to assessing the direct channels by which the ESF program affects individual health, this study aimed to contribute by testing whether there was an indirect effect coming from the density of the ESF teams within the state or the metropolitan area in which the individual lived.

To do so, the measure of coverage was disaggregated into two direct dimensions and one indirect dimension, as follows:

*ESF registration*: This is a binary variable (dummy) that equals one if the household was registered in the ESF program and zero otherwise, according to the individual’s answer in the PNS 2013. This variable is a measure of coverage and captures the direct effect of being assisted by an ESF team.

*ESF visits*: This is the number of annual visits received by the household. The survey asked how often an ESF agent visited the household in the 12 months prior to the survey, and the number of visits was calculated according to the option chosen by the respondent. This variable was included to evaluate whether the intensity of primary care coverage had any relationship with the laboratory results.

*ESF program supply:* This is the number of ESF teams by 10,000 inhabitants deployed to the metropolitan area or to the state in which the respondent dwelt in 2013 (source: Datasus [21]).

The answers from the PNS allowed us to include three main sets of control variables^5^:

Individual characteristics: sex, age, ethnicity, marital status, highest educational level (completed high school or completed college), individual health insurance, smoking status and practice of physical activity at least once a week.

Individual health conditions: obesity (individuals with a Body Mass Index > 30 kg/m^2^); waist circumference; a previous diagnosis of the following conditions: chronic disease, kidney failure, heart condition, diabetes, cancer and/or high blood pressure; and if the individual was hospitalized in the 12 months prior to the survey.

Household characteristics: household income per capita, participation in the federal cash transfer program (Bolsa Familia), and the presence of piped water, electric power, sanitation, and/or garbage collection. These covariates aimed to control for socioeconomic characteristics that affect health. The descriptive statistics of both coverage and control variables can be found in Tables S1 and S2 in the Additional File 1.

Additionally, the number of hospital beds per capita was included to control for the regional/local supply of health facilities (Source: Datasus [21]).

The final number of individuals tested was below the actual drawn number due to logistic difficulties, which required a poststratification procedure. Sampling weights were calculated for use of the laboratory exam dataset, given the poststratification procedures by sex, age, ethnicity, and educational level according to large regions, from the total sample of the PNS [18]. Fiocruz released the weights of the laboratory exams, which were used to weigh the standard deviations of the estimates [18].

The relationship between ESF program coverage and individual health was estimated through a probability model, in which the dependent variable presented the likelihood of an abnormal biomarker level. The probit model can be generically expressed as (1):

1 $$\Pr \left(y=1|X\right)=\Phi \left({\beta}_1 ESFregistration+{\beta}_2 ESFvisits+{\beta}_3 ESFsupply+ X\gamma \right)$$

where *y* is the binary variable equal to one if the result of the individual laboratory test is out of the reference range for the corresponding biomarker (Table S1 – Additional File 1) and zero otherwise, and *X* is the vector containing the set of controls. Φ(.) is the cumulative of the standard normal distribution.

## Results

Tables 1, 2, 3, 4 present the results for the likelihood of abnormalities in biomarkers, expressed as probit marginal effects.^6^ With the purpose of assessing both direct and indirect effects, the estimates were grouped by the four types of coverage. Specification I took the full sample into account and considered only household coverage (*ESF program registration* and *ESF visits*); Specification II considered only individuals that were tested in metropolitan areas with household coverage and metropolitan *ESF supply*, as defined in the previous section; Specification III took into account all individuals that were tested with household coverage and the corresponding state *ESF supply*; and finally, Specification IV considered the full sample and household coverage (as Spec. I) but replaced the regional health facilities with binary variables that identified the state or, if applicable, the metropolitan area the respondent dwelt in, with the purpose of capturing the specific regional effects.

**Table 1:** Results considering only household coverage (Spec. I)

	Leukopenia	Leukocytosis	Diabetes Mellitus	High Cholesterol	Kidney Failure	Low Platelets	Anemia	Arterial Hypertension
**ESF registration**	−0.0192**	− 0.0186**	− 0.00847	0.00287	− 0.0119**	− 0.0137**	−0.0216**	− 0.0162**
	**(0.00979)**	**(0.0094)**	(0.00707)	(0.00597)	**(0.00465)**	**(0.0056)**	**(0.00982)**	**(0.00797)**
**ESF visits**	0.00180*	0.000266	0.000454	−0.000382	− 0.000247	0.00141***	− 0.000893	− 0.00043
	(0.00095)	(0.0009)	(0.00068)	(0.00058)	(0.000483)	(0.0005)	(0.000975)	(0.000785)
**Health facilities**	−0.00404***	−0.0143***	− 0.0079***	−0.000729	− 0.00106*	−0.00103	0.000204	0.0026***
	(0.0012)	(0.00116)	(0.00088)	(0.00076)	(0.000574)	(0.00066)	(0.00123)	(0.001)
**Observations**	8831	8831	8831	8831	8831	8831	8831	8831

Weighted standard errors are in parentheses; *** *p* < 0.01, ** *p* < 0.05, * *p* < 0.1. All results were controlled for the individual characteristics (sex, age, ethnicity, marital status, educational level), individual health conditions (obesity, waist measure, previous diagnosis of chronic diseases, kidney condition, heart condition, diabetes, cancer, high blood pressure, hospitalized in the previous 12 months), household characteristics (presence of piped water, electric power, sanitation, garbage collection, household income per capita), whether the individual was covered by health insurance or not, if they are were Bolsa Familia recipient or not, if they participated in sports or not, and if they were a smoker or not.

**Table 2:** Results considering household coverage and metropolitan ESF supply (Spec. II)

	Leukopenia	Leukocytosis	Diabetes Mellitus	High Cholesterol	Kidney Failure	Low Platelets	Anemia	Arterial Hypertension
**ESF registration**	−0.0203*	− 0.0209*	− 0.0103	− 0.00515	−0.00877	− 0.0107	−0.00766	− 0.0129
	**(0.0118)**	**(0.0119)**	(0.00968)	(0.0087)	(0.00660)	(0.007)	(0.0137)	(0.0107)
**ESF visits**	−0.00088	0.000526	−7.67E-05	−0.000101	− 0.000493	− 0.00017	0.00173	0.000883
	(0.00137)	(0.0013)	(0.00107)	(0.00098)	(0.000819)	(0.0007)	(0.00155)	(0.00117)
**Metropolitan ESF supply**	0.00889	−0.0160	− 0.00341	− 0.00447	− 0.0168***	0.00706	− 0.0607***	−0.0302***
	(0.0102)	(0.0108)	(0.00872)	(0.00779)	**(0.00640)**	(0.006)	**(0.0122)**	**(0.0099)**
**Health facilities**	−0.00069	−0.0059***	−0.0049***	− 0.00043	0.000259	− 0.00003	0.00592***	− 0.000553
	(0.0012)	(0.00124)	(0.00104)	(0.00095)	(0.000716)	(0.00071)	(0.00145)	(0.00117)
**Observations**	3840	3840	3840	3815	3829	3840	3840	3840

Weighted standard errors are in parentheses; *** *p* < 0.01, ** *p* < 0.05, * *p* < 0.1. All results were controlled for the individual characteristics (sex, age, ethnicity, marital status, educational level), individual health conditions (obesity, waist measure, previous diagnosis of chronic diseases, kidney condition, heart condition, diabetes, cancer, high blood pressure, hospitalized in the previous 12 months), household characteristics (presence of piped water, electric power, sanitation, garbage collection, household income per capita), whether the individual was covered by health insurance or not, if they were a Bolsa Familia recipient or not, if they participated in sports or not, and if they were a smoker or not. The metropolitan ESF supply was measured as the number of local ESF teams by 10,000 inhabitants at the metropolitan level.

**Table 3:** Results considering household coverage and state ESF supply (Spec. III)

	Leukopenia	Leukocytosis	Diabetes Mellitus	High Cholesterol	Kidney Failure	Low Platelets	Anemia	Arterial Hypertension
**ESF registration**	−0.0209**	− 0.0187**	− 0.0078	0.00236	− 0.0118**	− 0.0137**	− 0.0218**	−0.0148*
	**(0.0098)**	**(0.0094)**	(0.00708)	(0.00599)	**(0.00465)**	**(0.0056)**	**(0.00983)**	**(0.00797)**
**ESF visits**	0.00151	0.000251	0.000139	−0.000447	−0.000222	0.00141***	−0.00094	− 0.000211
	(0.00095)	(0.0009)	(0.00068)	(0.00058)	(0.000486)	(0.0005)	(0.000979)	(0.00078)
**State ESF supply**	0.0226***	0.00114	−0.00771*	0.00484	−0.00143	−7.07E-05	0.00337	−0.0161***
	(0.0062)	(0.0059)	**(0.00458)**	(0.0039)	(0.00308)	(0.0034)	(0.00636)	**(0.00528)**
**Health facilities**	−0.00329***	− 0.0142***	− 0.0081***	−0.000552	− 0.00111*	−0.00103	0.000328	0.00197*
	(0.0012)	(0.00117)	(0.00089)	(0.00077)	(0.000584)	(0.00067)	(0.00125)	(0.00102)
**Observations**	8831	8831	8831	8831	8831	8831	8831	8831

Weighted standard errors are in parentheses; *** *p* < 0.01, ** *p* < 0.05, * *p* < 0.1. All results were controlled for the individual characteristics (sex, age, ethnicity, marital status, educational level), individual health conditions (obesity, waist measure, previous diagnosis of chronic diseases, kidney condition, heart condition, diabetes, cancer, high blood pressure, hospitalized in the previous 12 months), household characteristics (presence of piped water, electric power, sanitation, garbage collection, household income per capita), whether the individual was covered by health insurance or not, if they were a Bolsa Familia recipient or not, if they participated in sports or not, and if they were a smoker or not. The state ESF supply was measured as the number of ESF teams by 10,000 inhabitants at the state level.

**Table 4:** Results considering household coverage with state and metropolitan fixed effects (Spec. IV)

	Leukopenia	Leukocytosis	Diabetes Mellitus	High Cholesterol	Kidney Failure	Low Platelets	Anemia	Arterial Hypertension
**ESF registration**	−0.0141	− 0.00394	− 0.00248	0.00297	− 0.0101**	− 0.0141**	− 0.0135	− 0.0174**
	(0.00988)	(0.0092)	(0.00704)	(0.00607)	**(0.00480)**	**(0.0057)**	(0.0099)	**(0.0081)**
**ESF visits**	0.000666	−5.9E-05	0.000462	−0.00033	−0.000289	0.00126**	−0.000239	−0.000598
	(0.00097)	(0.0009)	(0.00069)	(0.0006)	(0.000513)	(0.0005)	(0.000994)	(0.000808)
**Observations**	8831	8831	8831	8831	8831	8831	8831	8831

Weighted standard errors are in parentheses; *** *p* < 0.01, ** *p* < 0.05, * *p* < 0.1. All results were controlled for the individual characteristics (sex, age, ethnicity, marital status, educational level), individual health conditions (obesity, waist measure, previous diagnosis of chronic diseases, kidney condition, heart condition, diabetes, cancer, high blood pressure, hospitalized in the previous 12 months), household characteristics (presence of piped water, electric power, sanitation, garbage collection, household income per capita), whether the individual was covered by health insurance or not, if they were a Bolsa Familia recipient or not, if they practiced sports or not, and if they were a smoker or not. Health facilities were replaced with state and metropolitan binary variables.

Table 1 exhibits the results for Specification I. Except for diabetes mellitus and high cholesterol, being registered in the ESF program was associated with a lower likelihood of abnormal ranges of biomarkers linked to all comorbidities. Kidney failure had the smallest marginal effect, i.e., the likelihood of observing an abnormal biomarker for this health condition was approximately 1.19 p.p. lower in registered individuals than in nonregistered individuals, whereas the likelihood reached 2.16 p.p. for anemia. Although being registered in the ESF program was negatively associated with the likelihood of thrombocytopenia (marginal effect of − 1.37 p.p.), the number of visits, on the other hand, was positively associated with the likelihood of this comorbidity.

The spillover effect at the metropolitan level (Table 2) was statistically significant for kidney failure, anemia and arterial hypertension. On average, the increase of one ESF team per 10 thousand inhabitants in metropolitan areas decreased the likelihood of kidney failure by 1.68 p.p., the likelihood of anemia by 6.07 p.p. and the likelihood of arterial hypertension by 3.02 p.p. Under this specification, the household coverage effect disappeared for all conditions, except leukopenia and leukocytosis at a 10% significance level.

Except for diabetes mellitus and abnormal cholesterol levels, registration in the ESF program remained significant for all health conditions when the spillover effect at the state level was included (Table 3), with magnitudes similar to Specification I (Table 1). Additionally, the positive association between visits and the likelihood of thrombocytopenia remained. However, the ESF supply at the state level proved to be significant only for arterial hypertension (marginal effect of − 1.61 p.p.) and for diabetes mellitus (− 0.7 p.p at a 10% significance level). The association between ESF supply at the state level and leukopenia was positive and significant at the 1% level.

When the health facilities were replaced with metropolitan and state-specific effects (Table 4), the negative association between household coverage remained statistically significant for kidney failure, thrombocytopenia and arterial hypertension, as well as the positive association between thrombocytopenia and the number of visits.

Finally, diabetes mellitus was the only comorbidity whose decrease in likelihood was robustly linked to health facilities because the effect of hospital bed density was negative and statistically significant at both the state and metropolitan levels. The other health conditions did not present a clear relationship with this variable, whereas anemia (in metropolitan areas) and arterial hypertension both presented a positive association.

## Discussion

Leukopenia and leukocytosis can arise as a result of several diseases, and it is difficult to assess the channel through which the ESF program acts on the corresponding biomarkers. The results suggest that household registration is negatively correlated with the likelihood of observing such conditions. Because hospitalizations arising from acute infectious diseases, such as tuberculosis and syphilis, are avoidable through primary care services [33], one possible explanation for the obtained results is that preventive actions in long-term primary care contribute to a decrease in the incidence of infections and to the maintenance of immunity in general.

There is well-documented evidence of altered cholesterol levels in the Brazilian adult population, mainly among women, elderly people and less educated people [36]. Additionally, the prevalence of diabetes among the Brazilian adult population is approximately 10% [37]. The results for diabetes mellitus and cholesterol revealed no direct effect of the ESF program on the likelihood of abnormal levels of their corresponding biomarkers. On the other hand, the presence of health facilities seemed to be more important than primary care services for diabetes, as the effect of hospital bed density was negative and significant for all specifications regarding this disease. The indirect effect appeared only for diabetes at the state level, with a 10% significance level. These results seem disappointing, as one would expect that the ESF directly impacts diabetes and cholesterol though educative actions, since these two conditions have an association with poor eating habits. There is accumulated evidence [11] that the intensity of ESF services can reduce premature mortality from complications of hypertension, heart failure, cerebrovascular diseases, and diabetes. This combination of results seems to indicate that the development of diabetes and hypercholesterolemia is multifactorial, mixing environmental and genetic causes that are not addressed by the ESF program. Nevertheless, although the ESF program is unable to mitigate both conditions, the program is effective in reducing some of their most serious consequences in the long run, such as mortality.

Kidney failure and anemia are both more prevalent among elderly people and the low-income population in Brazil [19, 38]. The results suggest a negative association between the likelihood of such comorbidities and ESF program coverage, which signals that the program has been successful in addressing these comorbidity-vulnerable populations.

The ESF program dynamics in the metropolitan regions seemed to be quite different than the average because individual coverage was not significant for all comorbidities within such regional constituencies, except leukopenia and leukocytosis at a 10% significance level. On the other hand, the spillover effects seemed to be negatively associated with kidney failure, anemia and high blood pressure in the metropolitan areas. The reasons for these results deserve deeper investigation. It is possible that the dynamics of the ESF in great urban clusters are different from those in smaller cities and include integrated actions with other agents that are not directly linked to primary care coverage.

The number of annual visits was positively associated with thrombocytopenia, which was against the expected effect. Although a low blood platelet count is not preventable in most cases, it can denote/signal several health problems [39]—a deficiency of vitamin B12, liver diseases (cirrhosis), spleen problems, alcohol abuse, hepatitis C, some types of leukemia, or changes in bone marrow. It is possible that ESF teams more often visit households whose residents suffer from chronic diseases associated with this biomarker, which would explain the obtained effects, but this hypothesis deserves more investigation.

The spillover effect was strong for arterial hypertension, both at the metropolitan and state levels. Except in metropolitan areas, being registered in the ESF program was also important for avoiding hypertension, reducing the likelihood of abnormal biomarkers. This result is in accordance with recent evidence that the ESF program reduces mortality from complications of hypertension, including cerebrovascular diseases [8, 11].

It is important to emphasize that, given the empirical strategy adopted in this study and the limitation of the datasets, a statistically significant result does not necessarily mean causality because the exposure and the outcome measures were taken at the same time (2013). It is wiser to interpret the results as statistically significant contemporary relationships. Nonetheless, the findings reveal important connections to guide public policies on primary health care.

Unfortunately, the sample design of the PNS 2013 did not allow us to identify all the municipalities where the data were collected, but only the federation unit and the metropolitan region, if applicable. For a more refined study of the relationship between the density of ESF teams and the effects on individual health, it would be necessary to identify the location of each household; this would be an interesting next step for this research. Despite this limitation, this paper contributes to the field as a first investigation and could shed light on important policy priorities for primary health care in Brazil.

## Conclusions

Rather than having a unique objective, the Family Health Strategy has a set of multiple actions, as the most important primary care program in Brazil, from preventing infant mortality to referring patients for more complex procedures in outpatient clinics and hospitals. In this sense, it is not easy to draw objective policy conclusions from the results mentioned above. They reveal a complex combination of direct and indirect effects, with different linkages according to the type of coverage, the intensity of care and the dwelling region. Nonetheless, the results here allow us to state that increasing primary care coverage is a promising strategy to address important comorbidities in low- to middle-income countries. Moreover, the presence of indirect effects (spillover) suggests that health authorities should treat metropolitan areas in a different fashion, developing specific and coordinated actions among the municipalities that belong to them.

## Supplementary Information

**Additional file 1.**

## Data Availability

The data that support the findings of this study are openly available for download from the Brazilian Ministry of Health/Datasus [https://datasus.saude.gov.br/transferencia-de-arquivos/], from the Brazilian Institute of Geography and Statistics/PNS 2013 [https://www.ibge.gov.br/estatisticas/sociais/saude/9160-pesquisa-nacional-de-saude.html?=&t=downloads] and from the Oswaldo Cruz foundation (Fiocruz) [https://www.pns.icict.fiocruz.br/bases-de-dados/]. The final/combined dataset (in Stata format) is available from the corresponding author on request.

## Abbreviations

BMI: Body mass index
ESF: Estratégia de saúde na família (family health strategy).
Fiocruz: Osvaldo Cruz foundation
GFR: Glomerular filtration rate
HDL: High density lipoprotein
IBGE: Brazilian institute of geography and statistics
LDL: Low density lipoprotein
PNAD: Pesquisa nacional por amostra de domicílio (annual national household sample survey)
PNS: Pesquisa nacional de saúde (national health survey)

## Footnotes

1 For details on the sample design of the PNS 2013, see [17].

2 The second edition of the PNS was carried out in 2019, but no laboratory tests were conducted in this edition.

3 All data used in this study were made publicly available on the internet [20–22] by the institutions responsible for collecting the data, so no ethical approval was required. The Brazilian legislation (Resolution 510, of April 7, 2016, from the National Health Council/Health Ministry) establishes that studies that use information with public access will not be registered or evaluated by the Research Ethics Committee. This study fits into that category.

4 While the PNS does not identify the surveyed municipalities, its design allows the identification of whether a household is located in a metropolitan area. As created by the law, metropolitan areas are clusters of urban cities that coordinate joint actions of development and set up unified policies to address common problems. According to the last Brazilian National Census (2010), approximately 40% of the Brazilian population dwelt in 21 metropolitan areas around the state capitals. Among the 8900 individual lab tests, there was a subsample of 3879 collected across 21 identifiable metropolitan areas.

5 Nonquantitative characteristics were measured through binary variables that assumed value one if the individual fulfilled the characteristic and zero otherwise.

6 The marginal effect means the change in the likelihood of a disease/comorbidity given a small change in the explicative variable. For binary explicative variables, the marginal effect must be interpreted as the change in the likelihood of disease if the explicative variable changes from 0 to 1.
