# Supplementary material for: Primary care coverage and individual health: evidence from a likelihood model using biomarkers in Brazil
Source: BMC Health Serv Res. 2021 Dec 4;21:1300. doi: 10.1186/s12913-021-07329-9 (PMC8642960; doi:10.1186/s12913-021-07329-9)
Supplement: Supplementary file 1 — Additional file 1. [file 12913_2021_7329_MOESM1_ESM.docx]

| Condition | Biomarker and range for abnormality |
| --- | --- |
| Leukopenia | White blood cells < 4,500 mm^3^ |
| Leukocytosis | White blood cells > 11,000 mm^3^ |
| Diabetes Mellitus | Estimated average blood glucose > 126 mg/dL; Glycated Hemoglobin > 6.5% |
| Hypercholesterolemia | Total cholesterol > 200 mg/dL for adults and > 170 mg/dL for teenagers between 18 and 19 years of age; LDL > 130 mg/dL for adults and > 110 mg/dL for teenagers between 18 and 19 years of age; HDL < 40 mg/dL |
| Kidney failure | Serum creatinine > 1.20 mg/dL and Glomerular Filtration Rate (GRF) < 60 mL/min/1.73 m^2^ |
| Thrombocytopenia | Platelets < 150,000 mm^3^ |
| Anemia | Hemoglobin < 14 g/dL for men and < 12 g/dL for women (< 11.5 g/dL if pregnant). |
| Blood pressure | Systolic blood pressure > 139 mmHg; Diastolic blood pressure > 89 mmHg. |

**Chart S1***.* Criteria for abnormal results according to the condition

Sources: [1-5]. When the sources did not indicate a consensual range, the less stringent one was adopted.


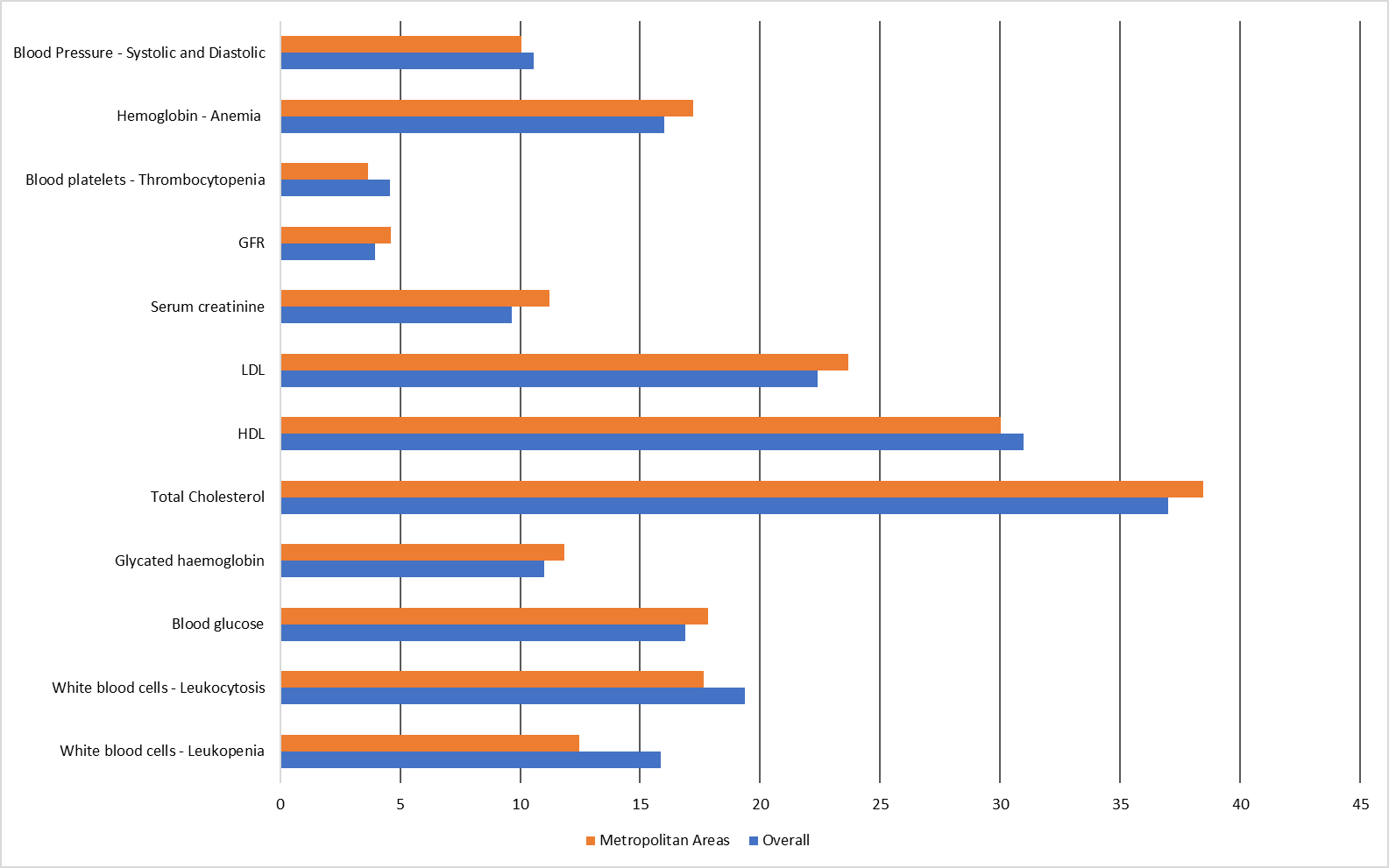
**Chart S2.** Abnormal Results in Laboratory Tests, in %

Source: National Health Survey 2013 [6] - Total of 8,952 lab tests, 3,879 in metropolitan areas

**Table S1.** Binary control variables, in %

| **(%)** | **Overall** | **Metropolitan areas** |
| --- | --- | --- |
| Registered in the ESF program | 58.09 | 48.59 |
| Female | 58.39 | 60.07 |
| Married | 41.76 | 40.06 |
| Separated | 2.42 | 2.93 |
| Divorced | 4.64 | 5.1 |
| Widowed | 7.65 | 8.14 |
| Single | 43.5 | 43.74 |
| White | 36.89 | 39.36 |
| Brown | 52.21 | 49.16 |
| Black | 9.2 | 9.84 |
| Asian | 0.92 | 0.95 |
| Indigenous | 0.73 | 0.64 |
| Obesity | 22.75 | 24.03 |
| Some physical activity | 98.91 | 98.81 |
| Federal grant (Bolsa Familia) recipient | 13.52 | 10.29 |
| Health insurance | 23.27 | 31.45 |
| Previous diagnosis of hypertension | 23.49 | 24.23 |
| Previous diagnosis of diabetes | 6.71 | 7.81 |
| Previous diagnosis of dengue | 22.78 | 22.56 |
| Smoker | 14.44 | 12.66 |
| Previous diagnosis of cancer | 1.75 | 2.09 |
| Previous diagnosis of a heart condition | 4.5 | 4.51 |
| Previous diagnosis of a kidney condition | 1.59 | 1.37 |
| High school education | 29.79 | 34.6 |
| College education | 10.26 | 12.94 |
| Household piped water supply | 73.97 | 84.84 |
| Household sanitation | 39.53 | 56.92 |
| Household garbage collection | 75.7 | 87.55 |
| Household electricity power | 98.58 | 99.66 |

Source: National Health Survey 2013 [6] - Total of 8,952 lab tests

**Table S2.** Descriptive statistics of nonbinary variables

|  | **Obs.** | **Mean** | **Sd. Dev** | **Min** | **Max** |
| --- | --- | --- | --- | --- | --- |
| Overall |  |  |  |  |  |
| ESF visits (N) | 8952 | 3.89 | 5.11 | 0 | 12 |
| Body Mass Index (kg/m²) | 8856 | 26.57 | 5.1 | 13.11 | 61.35 |
| Waist circumference (cm) | 8853 | 91.12 | 13.13 | 50 | 149.7 |
| Age (years) | 8952 | 46.85 | 16.45 | 18 | 104 |
| Household income per capita (BRL/month) | 8915 | 643.87 | 801.19 | 44.17 | 13833 |
| Metropolitan Areas |  |  |  |  |  |
| ESF visits (N) | 3879 | 2.39 | 4.27 | 0 | 12 |
| Body Mass Index (kg/m²) | 3845 | 26.83 | 5.1 | 13.1 | 51.3 |
| Waist circumference (cm) | 3845 | 91.57 | 13.34 | 50 | 145 |
| Age (years) | 3879 | 47.05 | 16.43 | 18 | 104 |
| Household income per capita (BRL/month) | 3875 | 808.41 | 1081.251 | 50 | 13833 |

Source: PNS 2013 [6]; calculated by the authors.

**References/Sources**

1. SBC. Nova diretriz de hipertensão arterial traz mudanças no diagnóstico e tratamento. Sociedade Brasileira de Cardiologia. 2020. https://www.portal.cardiol.br/post/nova-diretriz-de-hipertensão-arterial-traz-mudanças-no-diagnóstico-e-tratamento. Accessed 3 Mar 2021.

2. ANAD. Associação Nacional de Atenção ao Diabetes. 2018. https://www.anad.org.br/posicionamento-oficial-sbd-sbpc-ml-sbem-e-fenad-2017-2018/. Accessed 14 Oct. 2021.

3. Stasi R. How to approach thrombocytopenia. Hematology Am Soc Hematol Educ Program. 2012;2012:191-7.

4. Balarajan Y, Ramakrishnan U, Ozaltin E, Shankar AH, Subramanian SV. Anaemia in low-income and middle-income countries. Lancet. 2011;378:2123-35.

5. Malta DC, Machado Í E, Pereira CA, Figueiredo AW, Aguiar LK, Almeida WDS, et al. Evaluation of renal function in the Brazilian adult population, according to laboratory criteria from the national health survey. Rev Bras Epidemiol. 2019;22Suppl 02:E190010.supl.2.

6. PNS. Pesquisa nacional de Saúde [data set]. Instituto Brasileiro de Geografia e estatística. 2013. https://www.ibge.gov.br/estatisticas/sociais/saude/9160-pesquisa-nacional-de-saude.html?=&t=downloads. Accessed 25 Feb 2021.
